# Intraneural or extraneural ganglion cysts as a cause of cubital tunnel syndrome: A retrospective observational study

**DOI:** 10.3389/fneur.2022.921811

**Published:** 2022-08-05

**Authors:** Ainizier Yalikun, Maimaiaili Yushan, Yimurang Hamiti, Cheng Lu, Aihemaitijiang Yusufu

**Affiliations:** Department of Microrepair and Reconstructive Surgery, The First Affiliated Hospital of Xinjiang Medical University, Urumqi, China

**Keywords:** anterior subcutaneous transposition (AST), cubital tunnel syndrome, ganglion cysts, surgical management, ulnar nerve

## Abstract

**Purpose:**

Cubital tunnel syndrome caused by ganglion cysts has rarely been reported. The purpose of this study was to evaluate the surgical treatment outcomes of a patient diagnosed with cubital tunnel syndrome caused by intraneural or extraneural cysts and to summarize our experience.

**Method:**

In total, 34 patients were evaluated retrospectively from January 2011 to January 2020 with a follow-up of more than 24 months. Preoperative data, such as demographic data, clinical symptoms, physical examination findings, and laboratory tests, were all recorded and pre-operative and post-operative data were compared. The function was evaluated by the modified Bishop scoring system and the McGowan grade at the last follow-up.

**Results:**

Improvement of interosseous muscle strength, the Visual Analog Scale (VAS), 2-point discrimination (2-PD), electromyogram (EMG) result, Wartenberg sign, claw hand, and weakness could be clearly observed in all patients. Extraneural cysts were completely removed and the pedicles of the cysts were ligated. Intraneural cysts were incised and drained, and part of their cyst walls were removed using a microsurgical technique. All patients underwent anterior subcutaneous transposition (AST). At the last follow-up, McGowan's (0-IIa) grade increased from seven patients (20.6%) preoperatively to 27 patients (79.4%); the excellent and good rate according to the modified Bishop scoring system was 82.4% (28 patients), and all patients had no symptoms of recurrence after surgery.

**Conclusion:**

The treatment of cubital tunnel syndrome caused by intraneural or extraneural cysts achieved good long-term results through extraneural cyst resection or intraneural cyst incision and drainage combined with subcutaneous transposition. Early diagnosis and surgical treatment are essential for the patient's postoperative recovery.

## Introduction

Cubital tunnel syndrome is the second most common type of compressive neuropathy in the upper extremities, surpassed only by carpal tunnel syndrome (1). A thorough understanding of the ulnar nerve (UN) anatomy and common sites of compression is required to determine the cause of the neuropathy and proper treatment (2). Cubital tunnel syndrome caused by elbow perineural ganglion cysts is rare in clinical practice. Some scholars reported that elbow ganglion cysts are the third most common causative factor associated with cubital tunnel syndrome. They estimated the prevalence rate of these cysts to be 8% in these patients (3).

Ganglion cysts are benign mucinous lesions that typically occur near joints and may affect neighboring nerves by their location and size (4). In the upper extremity, ganglion cysts occur most frequently in the ulnar nerve and may be intraneural or extraneural in location (5). According to a recent systematic review, neural cysts around the elbow comprise only 9% of all cases (6). However, there are still a few reports focusing on patients with cubital tunnel syndrome and ganglion cysts, and in addition, attempts to remove the cyst itself without recognizing the relationship between the cyst and the nerve in the treatment may lead to poor outcomes, such as permanent neurological deficits or neuropathic pain. Therefore, the purpose of this study is to present the microsurgical treatment and long-term postoperative outcomes of 34 consecutive cases of cubital tunnel syndrome caused by intraneural or extraneural cysts and to provide further clinical experience.

## Methods and materials

This is a retrospective observational study and has been approved by the Ethics Committee of the First Affiliated Hospital of Xinjiang Medical University. Patients with cubital tunnel syndrome due to ganglion cyst compression were treated at our institution from January 2011 to January 2020. The inclusion criteria were as follows: participants aged 18–65 years, who were diagnosed with cubital tunnel syndrome caused by ganglion cysts which was confirmed by postoperative pathological findings, and follow-up time ≥2 years after the operation. The exclusion criteria were as follows: (1) patients with suspected cervical spondylosis or thoracic outlet syndrome; (2) patients with carpal tunnel syndrome or other peripheral neuropathy; and (3) patients with severe cardiovascular or autoimmune diseases, poor compliance, and loss of follow-up. The patient was diagnosed with cubital tunnel syndrome by the clinical examination and electromyogram test. The clinical findings of the patient mainly included sensory dysfunction, muscle atrophy, and even motor dysfunction in the ulnar nerve innervation area, and the electromyogram results suggested that the motor nerve conduction velocity (MNVC) across the elbow of the ulnar nerve was significantly decreased. All patients routinely underwent anteroposterior and lateral X-rays of the elbow joint before surgery; 24 patients were found to have an elbow joint mass before surgery and underwent preoperative ultrasound and MRI. In this study, we recorded the preoperative clinical symptoms, physical examination, electromyogram (EMG), ultrasound, and MRI results of patients and compared them with the clinical and EMG results at the last follow-up, and the upper extremity function was evaluated by a modified Bishop scoring system (7) and McGowan grade (8).

### Surgical procedures

During the operation, an accurate incision was made on the medial side of the elbow, the ulnar nerve was carefully exposed and released microsurgically, all extraneural cysts were completely removed, and the cyst pedicle was ligated and sutured. For intraneural cysts, incision and drainage were performed to completely empty the cyst content and decrease the cyst's internal pressure. The cyst wall was partially removed to achieve complete decompression of the ulnar nerve in terms of avoidance of iatrogenic injury and potential cyst recurrence. All patients underwent subcutaneous transposition of the ulnar nerve after cyst removal to avoid potential recurrence of symptoms.

The McGowan classification (8) was used to grade the extent of ulnar neuropathy. A patient with symptoms without abnormal objective findings is classified as grade I. Grade II neuropathy is characterized by intrinsic weakness, with incomplete wasting and abnormality of moving 2-point discrimination (two PD). The McGowan grade II population is further subdivided on the basis of the extent of the motor. Grade IIA neuropathy is characterized by good intrinsic strength (four out of five) without detectable atrophy and grade IIB extremities demonstrate only fair intrinsic strength (three out of five) with intrinsic atrophy. Neuropathy with disabling symptoms, profound motor weakness, marked intrinsic atrophy, and profound sensory disturbances are classified as grade III. We used a modified McGowan classification such that Grade 0 included those with no sensory findings and no motor findings and that Grade 1 included those with sensory findings and no motor findings.

## Results

A total of 34 patients were included in this study, among whom 28 were men (82.4%). The mean age was 55.4 (36.4–61.5) years. The mean duration of symptoms was (5.4 ± 2.1) (2.0–11.9) months and the average follow-up was 49.4 ± 15.6 (24.4–89.5) months. There were five cases of the intraneural cyst, 29 cases of the extraneural cyst, and 32 patients (94.1%) with arthritis of the elbow joint indicated by X-ray. The average size of intraneural cysts was 1.7 × 1.1 cm (1.3–2.4 cm) × (0.6–1.3 cm), and the average size of extraneural cysts was 2.3 × 1.2 cm (1.2–4.8 cm) × (0.7–2.1 cm). The results of ultrasound showed that the cysts in or around the cubital tunnel were found in all patients, and all of them were further confirmed by MRI before operation (as seen in Table 1 for more details).

**Table 1:** Demographic data and clinical characteristics.

**Age, years**	**55.4 ±7.5**
Gender
Men, *n* (%)	28(82.4%)
Women, *n* (%)	6(17.6%)
Cyst type
Intraneural cyst, *n* (%)	5(14.7%)
Extraneural cyst, *n* (%)	29(85.3%)
Duration of symptoms, months	5.4 ± 2.1
Arthritis of elbow joint, *n* (%)	32(94.1%)
^*^Size of the intraneural cyst, cm	1.7 × 1.1 (1.3–2.4) × (0.6–1.3)
^*^Size of the extraneural cyst, cm	2.3 × 1.2 (1.2–4.8) × (0.7–2.1)
Follow-up, months	49.4 ± 15.6
Disease Severity
Grade I, *n* (%)	4(11.8%)
Grade IIa, *n* (%)	3(8.8%)
Grade IIb, *n* (%)	2(5.9%)
Grade III, *n* (%)	25(73.5%)

* Size of the cyst is described by the length of the cyst × the maximal axial diameter of the cyst.

All patients had improvement after surgery. A 2-PD was recovered from the preoperative average of (11.9 ± 2.3) mm to an average of (4.2 ± 1.1) mm. The improvement of the Visual Analog Scale (VAS) was observed in 33 patients (97.1%), from the preoperative 5.2 ± 1.3 (2–8) to 1.3 ± 0.7 (0–3). Interosseous muscle strength was improved in all patients. Interosseous muscle strength recovered ≥ Medical Research Council (MRC) grade 4 in 28 (82.4%) patients. MRC grades of any muscles <3 were defined as poor recovery. EMG was significantly improved after surgery in all patients. The MNCV of the ulnar nerve across the elbow recovered from 18.9 ± 9.3 (0–29.6) to 41.5 ± 4.3 (32.8–48.5) m/s. Patients with “claw hand” preoperatively decreased from 19 (55.9%) to 2 (5.9%), and patients with Wartenberg signs decreased from 26 (76.5%) to 4 (11.8%) (as seen in Table 2). At the last follow-up, McGowan's (0–IIa) grade increased from seven patients (20.6%) preoperatively to 27 patients (79.4%); the excellent and good rates according to the modified Bishop scoring system were 82.4% (28 patients), the fair rate was only 11.8% (four patients), and the poor rate was 5.9% (two patients). All patients had no symptoms of recurrence after surgery (as seen in Table 3).

**Table 2:** Physical examination findings.

	**Preoperative**	**Postoperative**	***p*-value**
Interosseous muscle strength	–	–	
1	9	0	
2	18	2	
3	4	4	
4	3	15	
5	0	13	
VAS pain	5.2 ± 1.3(2–8)	1.3 ± 0.7(0–3)	<0.001
MNCV, m/s	18.9 ± 9.3(0–29.6)	41.5 ± 4.3(32.8–48.5)	<0.001
2-PD, mm	11.9 ± 2.3	4.2 ± 1.1	<0.001
Claw hand, *n* (%)	19(55.9%)	2(5.9%)	
Wartenberg sign, *n* (%)	26(76.5%)	4(11.8%)	

VAS, visual analog scale/score; MNCV, motor nerve conduction velocity of the ulnar nerve at the elbow; 2-PD, 2-point discrimination.

**Table 3:** Preoperative and postoperative functional evaluation.

	**Preoperative**	**Postoperative**
Modified Bishop score
Poor	–	2(5.9%)
Fair	–	4(11.8%)
Good	–	7(20.5%)
Excellent	–	21(61.8%)
McGowan
0	0(0%)	17(50%)
I	4(11.8%)	2(5.9%)
IIa	3(8.8%)	8(23.5%)
IIb	2(5.9%)	3(8.8%)
III	25(73.5%)	4(11.8%)

## Discussion

Although potential ulnar nerve entrapment can occur at multiple points along its course, such as the arcade of Struthers, the medial intermuscular septum, the medial epicondyle, the cubital tunnel, and the deep flexor-pronator aponeurosis, the most common site of entrapment is the cubital tunnel (9–11). Cubital tunnel syndrome resulting from ulnar nerve compression by ganglion cysts in the cubital tunnel is rare (12).

In 1810, Beauchêne first described an intraneural cyst of the ulnar nerve at the elbow, which the author called a “cubital serous cyst.” This short description is that of the first known intraneural cyst, which involved the ulnar nerve at the elbow (13). Over the past two centuries, the pathogenetic mechanisms of intraneural cysts have remained controversial (6, 14). Several theories have been proposed by some scholars, such as the tumoral, degenerative, extraneural intrusion (15), and unifying articular (synovial) theories (16). The unifying articular (synovial) theory proposed by Spinner et al. is widely accepted. The fundamental principles of the unifying articular (synovial) theory are as follows (17–20): (1) cyst fluid dissects from a degenerative synovial joint along an articular branch; (2) following the path of least resistance, cyst fluid flows along the articular branch, typically into the parent nerve and/or other major nerves or terminal branches; and (3) pressure fluxes further alter cyst dimensions, configurations, and directionality. This theory can also be extrapolated to intraneural cysts elsewhere including the upper limb (18, 21) and applies equally to the extraneural cysts (21), except that their joint connections are through non-neural pedicles.

Some scholars believe that (3, 22, 23) there is a significant correlation between elbow ganglion cysts and arthritis. Allieu reported (24) that cubital tunnel ganglion cysts are more likely to occur in middle-aged men with a previous history of elbow trauma, which may be their most obvious causative factor. Studies reported (25) that 118 (64%) of 184 patients with cubital tunnel syndrome had arthritis, of which 19 patients with arthritis had cubital ganglion cysts. Tong et al. (26) reported 59 cases (57 patients) of cubital tunnel syndrome caused by ganglion cysts, of which 57 cases had osteoarthritis of the elbow joint. In a previous report, Kato (3) analyzed 38 patients with cubital tunnel syndrome caused by intraneural cysts, of which 37 patients (97%) showed arthritis in anteroposterior and lateral X-rays of the elbow. Of the 34 patients in this study, 32 patients (94.1%) had osteoarthritis, so we believe that there is a close relationship between elbow trauma and the formation of elbow ganglion cysts; in addition, elbow abnormalities may lead to an increase in the pressure of the elbow joint and synovial fluid production, which will more easily lead to the formation of cysts in the cubital tunnel. After the incision and drainage of the intraneural cysts and the complete resection of the extraneural cysts, all patients underwent subcutaneous transposition of the ulnar nerve and achieved satisfactory outcomes, with no cases of symptom recurrence after surgery. We believe that subcutaneous transposition creates a straight path for the nerve, which decreases tension and presumably enhances neural blood flow. It also helps to transfer the nerve from the limited cubital tunnel and keep it away from sites of mechanical irritation; exceptionally, if there is a recurrence of the cysts in the cubital tunnel, it is unable to compress the anterior transferred ulnar nerve. Ji Sup Hwang (27) insisted that AST has a preference over *in situ* decompression technique when the neuropathy is severe or when the nerve becomes unstable after simple decompression. Some scholars (28) compared the long-term revision rate of *in situ* ulnar nerve decompression with anterior subcutaneous transposition surgery for idiopathic cubital tunnel syndrome and came to the conclusion that, in the long-term follow-up, *in situ* decompression is seen to have a statistically significant higher reoperation rate compared with subcutaneous transposition.

**Figure 1:**
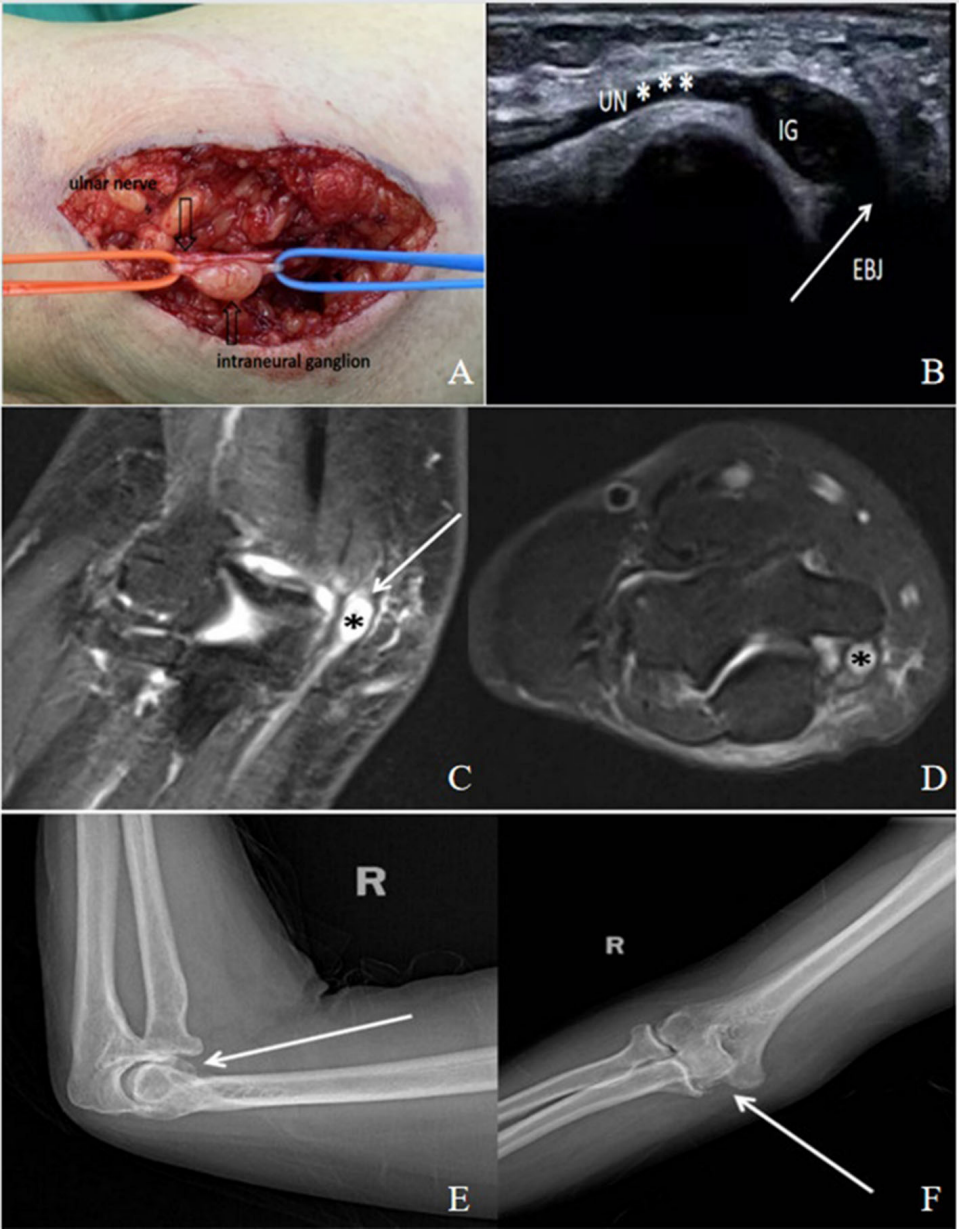
A 45-year-old male patient with cubital tunnel syndrome caused by intraneural ganglion (IG). **(A)** An intraneural ganglion (IG) was found within the ulnar nerve (UN*) in the cubital tunnel. **(B)** An ultrasound image shows an intraneural cyst extending along the ulnar nerve. EBJ: elbow joint. **(C,D)** There was a close link between the source of the intraneural cyst and the elbow joint, which is shown in the MRI (arrow). The intraneural ganglion (*) is shown as **(C)**, a high signal in T2STIR in sagittal view, and **(D)** a high signal in T2STIR in transverse view near the elbow joint. **(E,F)** Anteroposterior and lateral X-ray films of the elbow joint, showing the existence of elbow arthritis (arrow).

**Figure 2:**
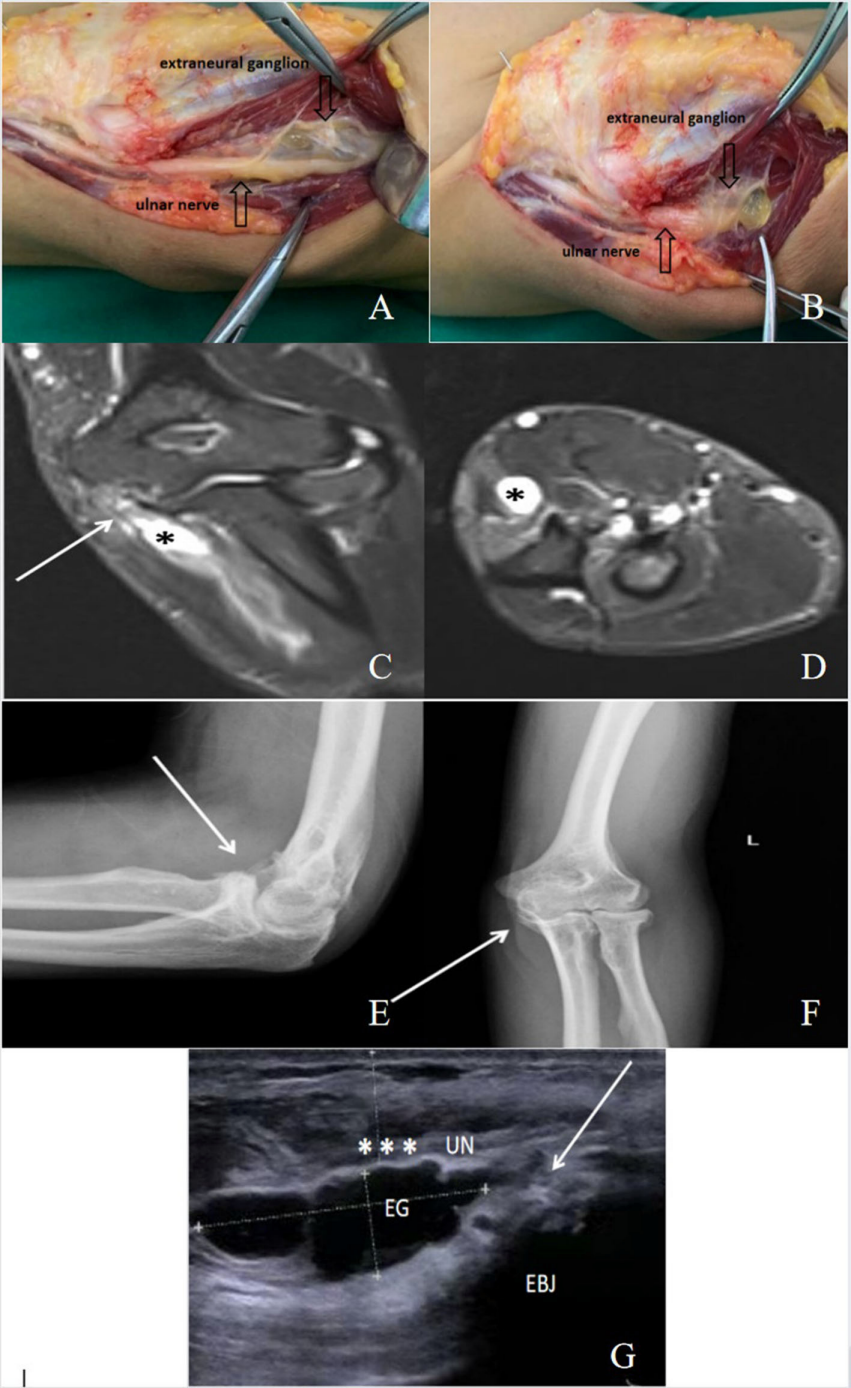
A 50-year-old male patient with cubital tunnel syndrome caused by extraneural ganglion (EG). **(A,B)** Intraoperative photograph of the extraneural ganglion, which is compressing and flattening the ulnar nerve in the cubital tunnel. **(C,D)** The extraneural ganglion (*) is shown as **(C)**, a high signal in T2STIR in sagittal view, and **(D)** a high signal in T2STIR in transverse view near the elbow joint. **(E,F)** Anteroposterior and lateral X-ray films of the elbow joint, showing the existence of elbow arthritis (arrow). **(G)** An ultrasound image showing an extraneural ganglion (EG) compressing the ulnar nerve (UN*). EBJ: elbow joint.

In another study, Spinner and Wang et al. (21) studied 86 patients with upper extremities intraneural cysts and found that a cystic articular branch (CAB) was present in only 20% of patients (17); a cystic articular branch was found in 40% of lower extremities intraneural cysts. They believed that the cystic articular branch exists in all intraneural cysts of the peroneal nerve and is easily ignored in imaging and intraoperative observation due to its relatively small size and the authors' unfamiliarity. In our study, in five patients with intraneural cysts, no definite CAB was observed during operation, but their ultrasound and MRI results suggested that there was a close link between the source of intraneural cysts and the elbow joint, so we considered the unifying articular (synovial) theory to be more acceptable. Prasad (29) and Spinner (30) proposed that the unifying articular (synovial) theory is also applicable to extraneural cysts, the site of origin of which depends on the site of the capsular tear and extends to any direction because of the lack of the limitation of the cystic articular branch, so there is uncertainty about its anatomical path (Figures 1, 2).

In this study, we performed incision and drainage of intraneural cysts by microsurgical instruments under a microscope. To fully decompress, part of the cyst wall was removed, the extraneural cyst was completely resected, and the root of the cyst was ligated. Allieu and Cenac (24) completely drained the upper extremities ganglion cysts with microsurgical instruments and removed part of the cyst wall. They pointed out that, to avoid iatrogenic injury to the nerve, resection of the part of the wall of the intraneural cysts under the microsurgical procedure also resulted in satisfactory outcomes. Desy (6) suggested that, to avoid postoperative neurological deterioration, incision of the cysts and removal of its contents should be performed for intraneural cysts rather than radical resection. Wayne (31) and Weyns (32) concluded that careful dissection is required for perineural cysts surgery, and the cystic articular branch of intraneural cysts or the pedicle of extraneural cysts should be resected and ligated. They recommend incision and drainage of intraneural cysts, and complete resection of extraneural cysts are particularly important for the treatment of perineural cysts. They believe that it helps to maximize the preservation of neurological function and reduce its risk of recurrence. According to the unifying articular (synovial) theory, intraneural cysts originate from joints rather than nerves, so complete resection of intraneural cysts is not required, but to prevent the recurrence of intraneural cysts, the cystic articular branch must be resected and ligated. Cobb et al. (33) performed incision and drainage of intraneural cysts at the peroneal nerve without cutting and ligating the cystic articular branch, which resulted in a recurrence rate of 30% after surgery, while the complete resection of intraneural cysts caused a recurrence rate of 29% and caused more serious damage to the peroneal nerve function. In our study, there was no symptom recurrence after the operation, and we may have unwittingly removed the cystic articular branch of the intraneural cyst during exposure and transposition of the ulnar nerve, thus eliminating the possibility of recurrence.

In the present study, two patients (5.9%) with poorly modified Bishop scores had the longest duration of symptoms, 8.5 and 11.9 months. Our findings are similar to those of recent studies (34–36), in which patients with short preoperative symptom duration had better postoperative recovery. Ming (37) proposed that the surgical outcome of intraneural cysts depends largely on the duration of symptoms and the degree of nerve damage, and early identification and timely intervention are usually related to good outcomes. Prognostic factors related to motor recovery may include the following: the duration of symptoms, the extent of the compression, the duration of the mass, and the length of the cyst. Tong (26) reported that smoking, shorter follow-up time, and older age were significant independent prognostic factors for higher McGowan grade after surgery. Overall, the average duration of symptoms in our study was 5.4 months; at the last follow-up, the number of patients with McGowan grade (0–IIa) increased from 7 (20.6%) to 27 (79.4%); the excellent and good rate according to the modified Bishop scoring system was 82.4% (28 patients); therefore, we believe that early diagnosis and surgical treatment are essential for the postoperative recovery of patients.

### Limitations of this study

The case number is relatively small to reach a strong conclusion because cubital tunnel syndrome caused by intraneural cysts is particularly rare; no recurrence of symptoms was found during the last follow-up in this study, which may be due to the ulnar nerve leaving its original location by subcutaneous transposition, so longer follow-up and examination of the cubital tunnel by ultrasound and MRI are needed to strengthen the further understanding of the disease.

## Conclusion

The treatment of cubital tunnel syndrome caused by intraneural or extraneural cysts in the elbow has achieved good long-term results through subcutaneous transposition combined with extraneural cyst resection and intraneural cyst incision and drainage. Early diagnosis and surgical treatment are essential for the patient's postoperative recovery.

## Data availability statement

The original contributions presented in the study are included in the article/supplementary material, further inquiries can be directed to the corresponding author/s.

## Ethics statement

The studies involving human participants were reviewed and approved by Ethical Committee of the first affiliated hospital of Xinjiang medical university. The patients/participants provided their written informed consent to participate in this study. Written informed consent was obtained from the individual(s) for the publication of any potentially identifiable images or data included in this article.

## Author contributions

AYa conducted the study, collected, analyzed, interpreted the data, and wrote the manuscript. MY designed the study, interpreted the data, and edited the manuscript. YH conducted the statistical analysis and interpreted the data. CL created and statistically analyzed the data. AYu planned the project and reviewed the manuscript. AYa and MY contributed equally to this study. All authors read and approved the final manuscript.

## Conflict of interest

The authors declare that the research was conducted in the absence of any commercial or financial relationships that could be construed as a potential conflict of interest.

## Publisher's note

All claims expressed in this article are solely those of the authors and do not necessarily represent those of their affiliated organizations, or those of the publisher, the editors and the reviewers. Any product that may be evaluated in this article, or claim that may be made by its manufacturer, is not guaranteed or endorsed by the publisher.
